# Hemorrhagic giant intra-abdominal desmoid tumor in twin pregnancy: a case report and literature review

**DOI:** 10.3389/fonc.2025.1575315

**Published:** 2025-11-24

**Authors:** Shizhao Wang, Zeqing Du, Lining Huang, Xiao Liu

**Affiliations:** 1 Department of Anesthesiology, The Second Hospital of Hebei Medical University, Shijiazhuang, China; 2 Department of Obstetrics and Gynecology, The Second Hospital of Hebei Medical University, Shijiazhuang, China; 3 Department of Medical Imaging, The Second Hospital of Hebei Medical University, Shijiazhuang, China

**Keywords:** desmoid tumor, soft tissue tumors, pregnancy, intra-abdominal, hemorrhage

## Abstract

**Background:**

Desmoid tumors (DTs) are rare, non-metastatic fibroblastic neoplasms known for their local invasiveness and slow growth. Their occurrence during pregnancy is rare, presenting unique diagnostic and therapeutic challenges, especially when complications like hemorrhage develop. Spontaneous hemorrhage in intra-abdominal DTs is an exceptionally rare complication.

**Case presentation:**

We report a 31-year-old woman, 32 + 4 weeks into a twin pregnancy, with a history of successful IVF-ET and routine prenatal care, who presented with acute left abdominal pain and signs of hemorrhagic shock. A computed tomography (CT) scan revealed a giant intra-abdominal mass (23.6×15.6×17.3 cm) with active bleeding. An emergency cesarean section and tumor resection were performed, which revealed a desmoid tumor (DT) invading the pancreas and transverse colon. Postoperative recovery was uneventful, and the patient was discharged in stable condition.

**Conclusion:**

This case underscores the importance of early multidisciplinary intervention in managing rare pregnancy-associated desmoid tumors (DT) complicated by hemorrhage. It emphasizes the necessity of heightened clinical suspicion in pregnant women presenting with acute abdominal pain.

## Introduction

Desmoid tumor (DT) is a rare fibroblastic neoplasm characterized by local invasiveness and the absence of metastatic potential (1). Although DT accounts for less than 3% of soft tissue tumors, its association with pregnancy is well-documented, especially in the abdominal wall. Intra-abdominal DT during pregnancy is extremely rare and often presents significant diagnostic and therapeutic challenges (2). We report a case of twin pregnancy complicated by a giant intra-abdominal DT with spontaneous hemorrhage, emphasizing the challenges in diagnosis and management.

## Clinical presentation

**Figure 1:**
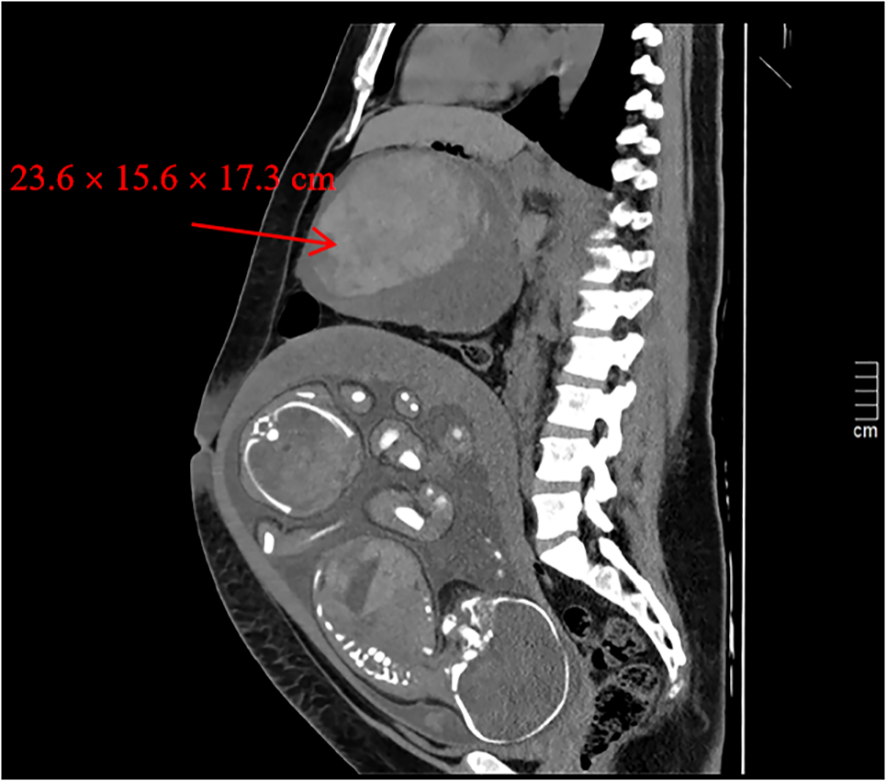
CT of the Abdomen and Pelvis revealed a large mixed-density mass (23.6 × 15.6 × 17.3 cm) in the upper abdominal cavity, with indistinct margins on the greater curvature of the gastric fundus (image resolution, 1004×881). The lower portion partially surrounds the transverse colon. The lesion is clearly demarcated f rom the liver, pancreas, and superior margin of the uterus. Findings suggest a hemorrhagic lesion.An enlar ged uterus containing twin fetuses is visible.

**Figure 2:**
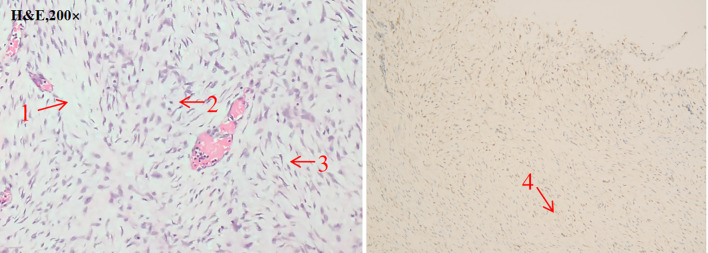
The tumor consists of bland spindle cells with abundant fibrotic stroma. Minimal mitotic activity is observed (H&E, 200×). Immunohistochemical analysis revealed Ki-67 (10%), Vimentin (+), and β-catenin (+). Arrows 1: M atrix composed of abundant interwoven collagen fibers. Arrows 2: Spindle/ovoid-shaped fibroblast cells. Arrows 3: Elongated cell nuclei without significant atypia. Arrows 4: β-catenin (+) area.

**Figure 3:**
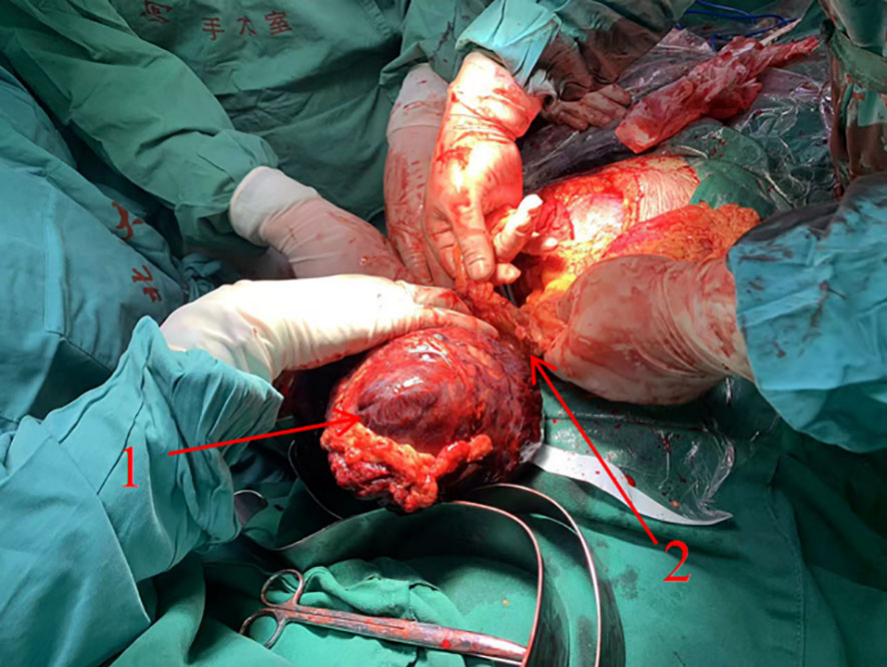
Intraoperative exploration revealed significant hemoperitoneum. A large intra-abdominal mass was located between the transverse colon and the greater curvature of the stomach, with rupture and active bleeding, surrounded by a larg e amount of blood clots. The mass was densely adherent to the left wall of the transverse colon, with a base approxi mately 2 cm in width, and was found to invade the pancreas. Arrows 1: The tumor. Arrows 2: Part of the transverse colon.

A 31-year-old woman, 32 + 4 weeks into a twin pregnancy, following successful IVF-ET and routine prenatal care, was admitted as an emergency on October 11, 2024. Her chief complaint was left abdominal pain, accompanied by nausea and vomiting, which had persisted for 9 hours. Upon admission, she was hypotensive (98/60 mmHg) and tachycardic (143 bpm). Initial laboratory tests revealed a hemoglobin level of 10.0 g/dL. An emergency CT scan identified a giant intra-abdominal mass (23.6×15.6×17.3 cm) (**Figure 1**) with active contrast extravasation, indicating active bleeding. An emergency cesarean section and tumor resection were performed via a midline laparotomy within 2 hours of admission. During surgery, the mass was found to be densely adhered to the wall of the transverse colon and involved the pancreas. The intraoperative blood loss was approximately 1600 mL, and the patient received 8 units of red blood cells and 800 mL of plasma. The postoperative pathology report, including both morphological and immunophenotypic analysis, confirmed the diagnosis of desmoid tumor. Immunohistochemical analysis showed Ki-67 (10%), Vimentin (+), and β-catenin (+) (**Figure 2**). The patient’s postoperative recovery was uneventful, and she was discharged in stable condition on October 18, 2024. Both neonates were transferred to the NICU due to prematurity. The first baby girl had a birth weight of 2000g, with Apgar scores of 7 and 8 at 1 and 5 minutes, respectively. The second baby girl weighed 1850g, with Apgar scores of 6 and 7 at 1 and 5 minutes, respectively. Both infants recovered well (**Figure 3**).

## Discussion

### Epidemiology of abdominal tumors in pregnancy

In pregnancies complicated by abdominal tumors, the majority (approximately 60-70%) are benign, with malignant tumors accounting for about 2-20%, and borderline tumors representing 10-15% of cases (3). Specifically, ovarian tumors, the most common abdominal tumors during pregnancy, are predominantly benign, including mature cystic teratomas and cystadenomas. Malignant tumors are most frequently represented by epithelial ovarian cancers and lymphomas, while borderline cases are typically serous or mucinous borderline tumors. This distribution is significant for clinical decision-making, as the high prevalence of benign lesions generally supports more conservative management approaches when possible. However, the potential for malignancy requires thorough evaluation of any persistent adnexal mass, particularly those with complex morphology on ultrasound or rising tumor markers.

### Desmoid tumor overview and pregnancy association

Desmoid tumors (DTs), also known as aggressive fibromatosis, are rare, clonal fibroblastic proliferations that arise in deep soft tissues. They are characterized by local invasiveness but lack metastatic potential. Approximately 15% of patients with familial adenomatous polyposis (FAP) develop one or more desmoid tumors during their lifetime (4). Although the incidence of DT is relatively low, it has been diagnosed more frequently during pregnancy in recent years, thanks to advances in imaging technology. The global incidence has increased from 2–4 per million to 5–6 per million (5).Pregnancy is considered a significant risk factor for DT, possibly due to hormonal changes such as elevated estrogen levels, mechanical stress from uterine expansion, and abnormal fibrous proliferation triggered by these factors (6–8),While DT during pregnancy is rare and typically found in the abdominal wall, intra-abdominal cases are even rarer (9).

### Unique features of our case

This case is unique because it involves a twin pregnancy complicated by a large intra-abdominal DT with hemorrhage, emphasizing the need for careful attention in diagnosing and treating massive abdominal masses during pregnancy. The physiological stress and volume changes associated with twin pregnancies may accelerate tumor progression and increase the risk of complications. Uterine enlargement and increased abdominal wall pressure could exacerbate tumor growth or trigger bleeding. In this case, the tumor was large, and the diagnosis was made only after hemorrhage occurred. This highlights the difficulty of diagnosing intra-abdominal tumors during pregnancy. Intra-abdominal bleeding is a rare complication of DT during pregnancy. This case represents the first report of pregnancy-related aggressive fibromatosis with hemorrhage. The occurrence of intra-abdominal hemorrhage typically indicates tumor rupture or vascular invasion, which can rapidly worsen the condition and pose a threat to both maternal and fetal life. In this case, acute hemorrhagic anemia and tumor rupture highlighted the high risk posed by the large DT and the potential harm it could cause during pregnancy. Early detection and timely intervention for intra-abdominal tumors are critical, particularly for rapidly identifying the bleeding source and implementing effective treatment.

### Literature search strategy

A comprehensive literature search was conducted to identify published cases of pregnancy-associated intra-abdominal desmoid tumors. The electronic databases PubMed/MEDLINE and Embase were searched for articles between January 2000 and December 2024. The search strategy utilized a combination of the following keywords and MeSH terms: “desmoid tumor,” “aggressive fibromatosis,” “pregnancy,” “gravidity,” “intra-abdominal,” “mesentery,” “retroperitoneum.” The inclusion criteria were: 1) case reports or case series, 2) patients diagnosed with an intra-abdominal desmoid tumor during pregnancy or in the immediate postpartum period, and 3) availability of clinical data on gestational age, management, and outcomes. Detailed statistical data are presented in **Table 1**.

**Table 1:** Summary of pregnancy-associated intra-abdominal desmoid tumors.

First author	Year	GA at diagnosis	Symptoms	Size (cm)	Management	Maternal-fetal outcome
Shlomo M Stemmer (7)	2024	2 months before becoming pregnant	a lump in her abdomen;rapid growth during pregnancy	3.0 × 2.3 × 3.0 cm	surgical resection at 25 weeks of pregnancy	Maternal uneventful recovery; Infant with normal Apgar scores
David Hanna (9)	2016	34 weeks gestation	Shortness of breath and abdominal pressure	12 cm	Observer; The mass rapid progression;Surgical resection	Maternal uneventful recovery; Healthy infant
Liquan Jin (10)	2020	32 weeks gestation	upper abdominal pain	21 × 12 cm^2^	fetal preservation treatment; Surgical resection after the delivery	Maternal uneventful recovery; Healthy infant
Eelco de Bree (11)	2013	3 weeks after vaginal delivery	persistent abdominal distension	33 × 29 × 8.5 cm	surgical resection	Maternal uneventful recovery; Healthy infant
Long Sun (12)	2007	Immediately after caesarean section	250 mL chylous ascites was found in her abdominal cavity during the operation	12.2 × 11.5 × 8.5 cm	surgical resection	One year after complete excision no recurrence; Healthy infant
Tankshali (13) PM	2011	During caesarean section	tumour mass showed a further volumetric increase	10.0 cm	surgical resection	Maternal uneventful recovery; Healthy infant
Siaudinyte Ieva (14)	2019	25 weeks of gestation	pain in the lower part of the abdomen, difficulties to sleep, and shortness of breath	9.0 × 9.0 × 9.8 cm	Observed;caesarean section at 32 weeks;surgical resection of a 40 × 30 × 25 cm	no postoperative complications and was discharged on postoperative day 10; Healthy infant
Present case	2024	32 + 4 weeks	abdominal pain	23.6 × 15.6 × 17.3 cm	surgical resection	Maternal uneventful recovery; Healthy infants

### Differential diagnosis of abdominal masses in pregnancy

The presentation of an acute abdominal mass during pregnancy requires a broad differential diagnosis. Although our case involves a desmoid tumor, several other neoplasms are more commonly encountered and should be considered. The diagnostic approach should integrate clinical history, tumor markers, imaging characteristics, and, ultimately, histopathological confirmation. **Table 2** provides a comparative overview of common abdominal tumors in pregnancy to aid clinical differentiation.

**Table 2:** Differential diagnosis of common abdominal tumors in pregnancy.

Tumor type	Typical clinical presentation	Key imaging features	Characteristic pathological & laboratory findings
Desmoid Tumor (This Case)	Acute or chronic abdominal pain; may present with hemorrhage or bowel obstruction.	Well-differentiated solid mass; moderate signal intensity on T2-weighted imaging (T2WI); strong late enhancement on MRI; no evidence of metastasis.	Immunohistochemistry (IHC): β-catenin (nuclear +), Vimentin (+). No elevated tumor markers.
Ovarian Cystadenoma (Serous/Mucinous)	Often discovered incidentally; large cysts may cause abdominal pain, pressure, or torsion.	Typically a unilocular or multilocular cystic mass with thin walls and minimal solid components.	Histology shows benign serous or mucinous epithelium. CA-125 levels may be mildly elevated.
Mature Cystic Teratoma (Dermoid Cyst)	Often asymptomatic and discovered incidentally; torsion may cause acute abdominal pain.	Complex cystic mass demonstrating fat-fluid levels, calcifications (teeth), and a Rokitansky protuberance on CT or MRI.	Contains mature tissues derived from all three germ layers, including skin, hair, and bone.
Gastrointestinal Stromal Tumor (GIST)	May present with gastrointestinal bleeding, abdominal pain, or as an incidental mass.	Typically appears as an exophytic, well-circumscribed mass arising from the stomach or small bowel wall; often heterogeneous due to necrosis.	IHC profile: CD117 (c-KIT) positive, DOG-1 positive.
Leiomyoma (Uterine Fibroid)	Usually identified before pregnancy; may cause pain, especially in cases of red degeneration.	Well-defined solid uterine mass; typically hypoechoic on ultrasound, with possible degenerative changes.	Histopathology shows a whorled pattern of smooth muscle cells. IHC: Desmin (+), SMA (+).
Phyllodes Tumor (Breast Primary)	Presents as a rapidly growing, palpable breast mass; rare cases may metastasize to abdominal viscera.	Imaging reveals a large, lobulated breast mass with cystic spaces; metastatic lesions in the abdomen appear solid.	Histopathology shows characteristic leaf-like architecture within the stromal component.

IHC, Immunohistochemistry; T2WI, T2-Weighted Imaging; US, Ultrasound; SMA, Smooth Muscle Actin.

### Imaging examination during pregnancy

Imaging modalities such as ultrasound, CT, and MRI play a crucial role in diagnosing desmoid tumors (DT) during pregnancy, helping to differentiate them from other causes of abdominal pain. DT typically manifests as a localized fibroblastic proliferation rather than a metastatic process, and imaging helps assess the extent and confinement of the lesion. Although DT has distinctive imaging characteristics, there are no pathognomonic symptoms or biomarkers; therefore, clinical findings must always be interpreted alongside imaging results. Ultrasound is the primary imaging tool during pregnancy and is valuable for initial tumor screening. Typical sonographic findings include an elliptical, well- or poorly defined solid soft-tissue mass with variable echogenicity. Most lesions exhibit posterior acoustic enhancement, and some show central necrosis or calcification. Color Doppler often demonstrates solid vascular flow with high resistance indices (RI > 0.70) (15). Recent reports suggest that contrast-enhanced ultrasound (CEUS) combined with superb microvascular imaging (SMI) can aid preoperative diagnosis of DT (16).

Because the relationship between the tumor, fetus, and placenta during pregnancy is complex, ultrasound assists in evaluating the tumor’s size, location, and proximity to surrounding structures. However, CT and MRI provide greater precision for deep-seated lesions. Enhanced CT and MRI are preferred for dynamic evaluation of intra-abdominal DTs, allowing detailed visualization of tumor boundaries, density, and anatomical relationships. Radiologic features typically include moderate signal intensity on T2-weighted imaging (T2WI), intermediate to high signal intensity on T1-weighted imaging (T1WI), strong late-phase enhancement on MRI, and elevated average ADC values—all consistent with aggressive fibromatosis. Arterial penetration signs on contrast-enhanced CT (CE-CT) and high ADC values are characteristic findings that assist in differentiating DT from other masses, reducing the need for invasive diagnostic procedures (17).

In this case, the fact that a large tumor went undetected during routine second-trimester prenatal ultrasound underscores the diagnostic limitations of imaging in pregnancy. Several factors likely contributed:1.The tumor’s upper abdominal location may have been obscured by the acoustic shadow of the gravid uterus.2.As a solid lesion, its echogenicity may not have contrasted sufficiently with adjacent mesenteric or visceral tissues.3.The acute clinical deterioration suggests rapid intratumoral hemorrhage during the third trimester, implying that the tumor may have been much smaller or asymptomatic earlier. This case demonstrates that a negative routine ultrasound does not rule out a large intra-abdominal mass. Clinicians should maintain a high index of suspicion when pregnant patients present with unexplained or acute abdominal pain.

### Radiation risk assessment in pregnancy

When CT imaging is considered for a pregnant patient, a meticulous risk-benefit evaluation is essential. The estimated fetal radiation exposure from a standard maternal abdominopelvic CT scan ranges between 10 and 50 milligrays (mGy). According to the American College of Radiology (ACR) and the International Commission on Radiological Protection (ICRP), the risk of radiation-induced teratogenesis or neurodevelopmental impairment is negligible at fetal doses below 100 mGy. The risk of childhood cancer increases only slightly compared with the baseline population incidence (18). In the third trimester, the primary concern shifts from congenital malformations to a marginally elevated lifetime risk of childhood malignancy. Although this risk is minimal, it necessitates strict adherence to the “as low as reasonably achievable” (ALARA) principle (19). In the present case, where massive intra-abdominal hemorrhage posed an immediate life-threatening condition for both the mother and fetuses, the urgent need for rapid and precise localization of the bleeding source justified the use of CT imaging. The diagnostic value and life-saving potential of the scan clearly outweighed the minimal radiation-related risk (20).

### Histopathological biopsy remains the gold standard for definitive diagnosis.

In summary, imaging plays a pivotal role in the early detection of desmoid tumors during pregnancy, but the choice of modality must be tailored to the specific gestational stage to ensure maternal and fetal safety. When the tumor is large and complicated by bleeding, both diagnosis and management become significantly more challenging.

The final diagnosis is confirmed through histopathology. Mutations in CTNNB1 or APC genes can lead to abnormal intracellular accumulation of β-catenin, a hallmark of desmoid-type fibromatosis. Immunohistochemical analysis typically demonstrates nuclear β-catenin positivity, confirming the diagnosis (21). In this case, immunohistochemistry revealed Ki-67 (10%), Vimentin (+), and β-catenin (+). Tumor cells showed strong nuclear β-catenin expression and were positive for Vimentin, with a Ki-67 proliferation index of approximately 10%. Although molecular testing for CTNNB1 or APC mutations was not performed due to the emergent nature of the surgery, the combined histomorphological and immunohistochemical findings definitively established the diagnosis of desmoid-type fibromatosis (22).

### Treatment of DT during pregnancy

Management of desmoid tumors (DT) in pregnancy requires a comprehensive assessment of the tumor’s location, size, growth rate, symptoms, and the safety of both mother and fetus. Tumor progression and complications—such as hemorrhage or invasion of adjacent organs—are critical factors guiding treatment decisions.If the tumor is small and asymptomatic during early pregnancy, conservative observation is usually sufficient. However, in the later stages of pregnancy, particularly when complications such as hemorrhage occur, early surgical intervention is recommended, as in this case. Hormonal fluctuations during pregnancy may influence DT growth; in some cases, spontaneous regression or stabilization occurs (23). Nonetheless, tumors that enlarge rapidly or cause acute symptoms (e.g., pain, bleeding, or compression of nearby structures) warrant prompt surgical consideration (24).

For abdominal wall lesions that do not involve the uterus, resection can often be performed safely. Several reports describe successful removal of abdominal wall DTs during pregnancy followed by normal vaginal delivery (6, 7). However, if the tumor grows rapidly in late pregnancy (e.g., after 32 weeks) or complications arise—such as hemorrhage, intestinal obstruction, or organ compression—urgent surgery should be performed, regardless of tumor location. During surgery, meticulous tumor resection and protection of the fetus are paramount.

In this case, acute symptoms developed at 32 + 4 weeks, and imaging confirmed a large hemorrhagic tumor, leading to an emergency cesarean section combined with tumor resection. Large intra-abdominal tumors increase operative difficulty and maternal-fetal risk because of pressure effects on surrounding organs. Intraoperatively, the mass was found to adhere tightly to the transverse colon and pancreas, requiring careful dissection to preserve uterine integrity and adjacent structures. This highlights the importance of a multidisciplinary approach, involving obstetricians, surgeons, anesthesiologists, neonatologists, and critical care specialists to ensure optimal outcomes for both mother and fetus.

Postoperative care is equally vital. Continuous monitoring of vital signs, hemodynamics, and potential complications (infection, hemorrhage, or bowel dysfunction) is essential. Particular attention should be paid to anemia correction, infection prevention, and bowel function recovery, especially following large tumor resections. Because surgery and anesthesia may impact neonatal outcomes, newborns should be closely observed for Apgar scores, respiration, and other vital signs, and transferred to the NICU if needed.

Desmoid tumors have a recurrence rate of approximately 50%, making postoperative imaging surveillance critical—typically at 3 months post-surgery, then periodically thereafter (25). For patients presenting with preoperative hemorrhage, individualized follow-up plans based on pathological and immunohistochemical findings are essential to monitor recurrence or complications.

In this case, the patient recovered uneventfully, and no abnormalities were detected at 3-month follow-up. This underscores three major lessons: (1) Early recognition is crucial, though imaging diagnosis becomes more challenging when tumors are large or hemorrhagic. (2) Timely surgery can prevent further deterioration and may reveal that tumor behavior in pregnancy differs from non-pregnant states. (3) Comprehensive perioperative management—including coordinated multidisciplinary care—is key to ensuring maternal and fetal safety, particularly in complex cases involving multiple gestations, large tumors, or intra-abdominal bleeding.

### Alternative treatment approaches for abdominal tumors during pregnancy

Interventional and minimally invasive procedures may serve as valuable adjunctive options in managing abdominal tumors during pregnancy.For highly vascular or hemorrhagic tumors (such as the desmoid tumor in this case), arterial embolization can provide emergency hemostasis and reduce tumor size, creating better conditions for subsequent treatment. However, the potential radiation exposure to the fetus must be carefully assessed before proceeding (26). For large symptomatic cystic lesions, ultrasound-guided aspiration or drainage can provide temporary relief from compression-related symptoms. Nevertheless, this approach is largely palliative, as recurrence rates remain high (27). Laparoscopic surgery represents another minimally invasive option suitable for small, well-localized tumors during early pregnancy. It offers advantages such as reduced surgical trauma, shorter recovery time, and less postoperative pain. However, its use becomes increasingly limited in mid-to-late pregnancy due to restricted intra-abdominal space and the heightened risk of uterine injury (3). Conservative management with active surveillance remains the preferred strategy for asymptomatic or benign-appearing lesions, particularly for desmoid tumors known to undergo spontaneous regression postpartum.

In conclusion, management of abdominal tumors during pregnancy should be highly individualized. A multidisciplinary team approach—involving obstetricians, surgeons, radiologists, anesthesiologists, and neonatologists—is essential to comprehensively assess tumor characteristics, gestational age, and maternal-fetal risk. Such collaboration allows for prudent selection among open surgery, minimally invasive procedures, interventional techniques, or conservative observation to achieve the best possible outcomes for both mother and child.

## Conclusion

In summary, this case of twin pregnancy complicated by a massive intra-abdominal desmoid tumor (aggressive fibromatosis) with hemorrhage underscores the complex clinical challenges posed by DT during pregnancy. It highlights the importance of maintaining a high index of suspicion for rare causes of acute abdominal pain in pregnant patients.

Early diagnosis and timely multidisciplinary intervention are essential for improving maternal and fetal outcomes. As diagnostic technology and clinical expertise continue to advance, the management of pregnancy-associated desmoid tumors will become increasingly precise and individualized.

Through scientifically guided and patient-specific treatment plans, the progression of DT during pregnancy can be effectively controlled, ensuring both maternal safety and fetal well-being. Furthermore, postoperative care and regular follow-up remain critical to achieving optimal long-term outcomes and minimizing the risk of recurrence.

## Data Availability

The data supporting this review are derived from previously published studies, all of which are cited in the manuscript. Original case data are not publicly available to protect patient privacy, but are available from the corresponding author upon reasonable request.
